# Association of Admission Glucose‐to‐Lymphocyte Ratio With 90‐Day Functional Outcome in Patients With Acute Ischemic Stroke Treated With Intravenous Thrombolysis

**DOI:** 10.1002/brb3.71484

**Published:** 2026-05-11

**Authors:** Dong Zhang, Ying Zong, Xiaoyan Qin, Ruinan Ma, Zhizhang Li, Xiaoguang Zhang, Ying Ding, Yueying Bai, Liang Hu, Yunhua Yue

**Affiliations:** ^1^ Department of Neurology, Yangpu Hospital, School of Medicine Tongji University Shanghai China; ^2^ Department of Geriatrics, Yangpu Hospital, School of Medicine Tongji University Shanghai China; ^3^ Department of Biology, Morrissey College of Arts and Sciences Boston College Chestnut Hill Massachusetts USA

**Keywords:** acute ischemic stroke, biomarker, functional outcome, glucose‐to‐lymphocyte ratio, intravenous thrombolysis

## Abstract

**Background:**

The glucose‐to‐lymphocyte ratio (GLR), a composite biomarker reflecting metabolic and immune‐inflammatory status, has shown prognostic value in several clinical settings. However, its relevance in patients with acute ischemic stroke (AIS) undergoing intravenous thrombolysis (IVT) remains unclear. We investigated the association between admission GLR and poor functional outcome at 90 days in this population.

**Methods:**

In this retrospective single‐center study, consecutive patients with AIS treated with IVT between May 2016 and December 2025 were included. The primary outcome was poor functional outcome at 90 days, defined as a modified Rankin Scale score of 3–6. GLR was analyzed as both a continuous and a categorical variable. Multivariable logistic regression, inverse probability weighting, restricted cubic spline analysis, subgroup analyses, and sensitivity analyses were performed after multiple imputation for missing data.

**Results:**

Among 785 patients, 29.64% had poor functional outcome at 90 days. The optimal GLR cutoff was 4.137, with a pooled AUC of 0.623 (95% CI 0.578–0.665). In the fully adjusted model, GLR was independently associated with poor outcome both as a continuous variable (OR = 1.071, 95% CI 1.004–1.144, *p* = 0.038) and as a categorical variable (OR = 1.639, 95% CI 1.057–2.543, *p* = 0.027). The findings remained robust in an alternative lipid‐adjusted model and IPW analyses. RCS analysis suggested an overall linear association, whereas sensitivity analyses using an alternative mRS dichotomization were not statistically significant.

**Conclusions:**

Elevated admission GLR was independently associated with an increased risk of poor functional outcome at 90 days in patients with AIS undergoing IVT. GLR may provide useful supplementary prognostic information for early risk stratification, although its standalone discriminatory performance was modest.

## Graphical abstract

**Objective**: To evaluate whether admission glucose‐to‐lymphocyte ratio (GLR) is independently associated with poor 90‐day functional outcome in patients with acute ischemic stroke treated with intravenous thrombolysis.

**Design**: Retrospective single‐center study of consecutive patients with acute ischemic stroke treated with intravenous thrombolysis.

**Primary Outcome**: Poor functional outcome at 90 days, defined as modified Rankin Scale score 3–6.

**Analyses**: Multivariable logistic regression, multiple imputation, inverse probability weighting, restricted cubic spline analysis, sensitivity analysis, and internal validation.

**Results**: Among 785 patients, 29.64% had poor 90‐day functional outcome. Higher admission GLR was independently associated with poor outcome both as a continuous variable (OR = 1.071, 95% CI 1.004–1.144, *p* = 0.038) and as a categorical variable (OR = 1.639, 95% CI 1.057–2.543, *p* = 0.027). Restricted cubic spline analysis suggested a broadly linear association. The discriminatory performance of GLR alone was modest (AUC = 0.623, 95% CI 0.578–0.665).

**Conclusion**: Admission GLR was independently associated with poor 90‐day functional outcome in patients with acute ischemic stroke undergoing intravenous thrombolysis. GLR may provide supplementary prognostic information, although its standalone predictive utility is modest.

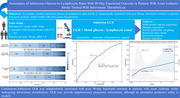

## 1 Introduction

Acute ischemic stroke (AIS) remains a leading cause of death and long‐term disability worldwide (Johnson et al. 2019). Although intravenous thrombolysis (IVT) is an established reperfusion therapy for eligible patients with AIS (Powers et al. 2018), a substantial proportion of patients still experience poor functional outcomes after treatment (Lees et al. 2010). In China, where the burden of stroke is particularly high, improving early risk stratification remains an important clinical priority (Ma et al. 2021).

AIS is characterized by a complex interplay among metabolic disturbance, systemic inflammation, and immune dysregulation (Capes et al. 2001; Chamorro et al. 2012; Iadecola and Anrather 2011; Kruyt et al. 2010; Urra et al. 2009). Acute hyperglycemia after stroke onset has been associated with larger infarct volume, blood–brain barrier disruption, oxidative stress, and poorer neurological recovery (Capes et al. 2001; Kruyt et al. 2010). Meanwhile, stroke‐related lymphopenia may reflect both the stress response and post‐stroke immunosuppression and has been linked to infection, secondary complications, and poor functional outcomes (Chamorro et al. 2012; Urra et al. 2009). These findings suggest that biomarkers integrating metabolic and immune‐inflammatory information may provide more comprehensive prognostic information than either indicator alone.

The glucose‐to‐lymphocyte ratio (GLR) is a readily available composite biomarker derived from routine laboratory testing that reflects both glycemic status and immune‐inflammatory response. Previous studies have suggested its prognostic value in cardiovascular disease and diabetes‐related populations (Lei et al. 2025; Lyu et al. 2025), and emerging evidence has linked GLR to stroke related complications such as stroke‐associated pneumonia (Zhou et al. 2025). However, the prognostic value of GLR in patients with AIS undergoing IVT remains insufficiently studied.

Therefore, this study aimed to investigate the association between admission GLR and poor functional outcome at 90 days in patients with AIS treated with IVT. We further evaluated the robustness, shape, and potential clinical relevance of this association using prespecified multivariable, weighted, subgroup, and sensitivity analyses.

## 2 Methods

### 2.1 Study Design and Population

This retrospective single‐center study included consecutive adult patients with AIS who underwent IVT at Yangpu Hospital, Tongji University School of Medicine, between May 2016 and December 2025. Eligible patients had imaging‐confirmed AIS and available admission laboratory data sufficient for calculation of the glucose‐to‐lymphocyte ratio. Patients undergoing bridging therapy were excluded from the primary analysis. Patients were also excluded if 90‐day functional outcome data were unavailable or if essential baseline data required for the primary analysis were missing beyond reasonable recovery through imputation.

The study was approved by the Ethics Committee of Yangpu Hospital, Tongji University School of Medicine (Approval No. LL‐2021‐LW‐003; February 22, 2021). All procedures were conducted in accordance with the Declaration of Helsinki, and patient confidentiality was strictly protected.

### 2.2 Data Collection and Variable Definition

Baseline demographic characteristics, vascular risk factors, blood pressure, Trial of Org 10172 in Acute Stroke Treatment (TOAST) classification (Adams et al. 1993), admission National Institutes of Health Stroke Scale (NIHSS) score (Brott et al. 1989), laboratory findings, and treatment‐related variables were extracted from the electronic medical record system. Symptomatic intracranial hemorrhage (sICH), defined according to the ECASS II criteria (Hacke et al. 1998), and early neurological deterioration (END), defined as an increase in NIHSS score of ≥2 points within 24 h (Seners et al. 2015), were recorded as secondary in hospital variables for sensitivity analyses. GLR was calculated as admission blood glucose divided by lymphocyte count (Zhou et al. 2025).

### 2.3 Outcome Assessment

The primary outcome was poor functional outcome at 90 days, defined as a modified Rankin Scale (mRS) score of 3–6. Functional outcome data were obtained through outpatient follow‐up or structured telephone interview. In a sensitivity analysis, an alternative dichotomization of functional outcome (mRS 0–1 vs. 2–6) was also examined.

### 2.4 Missing Data Handling

Missing baseline variables were handled using multiple imputation by chained equations. Twenty imputed datasets were generated. Overall, missingness was low across variables, with all missing rates below 5%. Derived variables such as GLR were recalculated within each imputed dataset from their imputed component variables rather than directly imputed. Pooled estimates were combined using Rubin's rules where appropriate.

### 2.5 Main Association Analyses

GLR was analyzed as both a continuous and a categorical variable. For categorical analyses, the optimal GLR cutoff was determined by receiver operating characteristic (ROC) analysis using the Youden index. The area under the ROC curve (AUC), sensitivity, specificity, and cutoff value were summarized across imputed datasets.

Multivariable logistic regression was used to assess the association between admission GLR and poor functional outcome at 90 days. GLR was modeled as a continuous variable, as a standardized variable per standard deviation increase, and as a categorical variable according to the ROC‐derived cutoff. Covariates included in the fully adjusted model were selected a priori on the basis of clinical relevance, prior literature, and potential confounding with both GLR and stroke outcome. To reduce potential multicollinearity among lipid parameters, total cholesterol (CHOL) was excluded from the main model, whereas low‐density lipoprotein cholesterol (LDL‐C) was retained.

### 2.6 Robustness, Sensitivity, and Weighted Analyses

Several additional analyses were performed to examine the robustness of the main findings. First, sensitivity models additionally adjusted for sICH or END. Second, an alternative lipid‐adjusted model was constructed by retaining CHOL while excluding LDL‐C. Third, GLR was analyzed by quartiles, and trend testing across quartiles was performed by assigning the median GLR value within each quartile and modeling this variable as a continuous term. Fourth, the primary outcome was alternatively defined as mRS 0–1 versus 2–6 in a sensitivity analysis.

To further address potential confounding, inverse probability weighting (IPW) was performed using the dichotomized GLR variable. Propensity scores were estimated from baseline covariates, and stabilized average treatment effect weights were calculated. Covariate balance before and after weighting was assessed using standardized mean differences (SMDs), with an absolute SMD < 0.1 considered indicative of adequate balance. Weighted logistic regression models with progressive adjustment for residual imbalance were fitted. Truncated weight analyses were also conducted as supplementary sensitivity analyses.

### 2.7 Shape, Subgroup, and Model Performance Analyses

Restricted cubic spline (RCS) analysis was performed within the fully adjusted model to evaluate the shape of the association between continuous GLR and poor outcome. Prespecified subgroup analyses were conducted according to age, sex, baseline NIHSS group, study period, previous stroke, atrial fibrillation, coronary artery disease, hypertension, and diabetes mellitus. Interaction tests were used to assess potential effect modification. Given the long study period, calendar time was incorporated into the adjusted models and subgroup analyses to partially account for potential temporal changes in stroke care.

The incremental prognostic value of GLR beyond the fully adjusted model was assessed by comparing discrimination and model fit, including changes in AUC, likelihood ratio testing, net reclassification improvement, and integrated discrimination improvement. Internal validation was performed using bootstrap resampling, and apparent calibration was assessed using calibration plots and the Brier score. Collinearity diagnostics were used to evaluate multicollinearity in the main adjusted model, and correlations among continuous covariates were assessed using Spearman correlation coefficients.

### 2.8 Statistical Analysis

Continuous variables are presented as mean ± standard deviation or median (interquartile range), as appropriate, and categorical variables as counts and percentages. Between‐group comparisons were performed using Student's *t*‐test, Mann–Whitney *U* test, chi‐square test, or Fisher's exact test, as appropriate. All tests were two‐sided, and a *p* value < 0.05 was considered statistically significant. Statistical analyses were performed using R software.

## 3 Results

### 3.1 Baseline Characteristics

**FIGURE 1:**
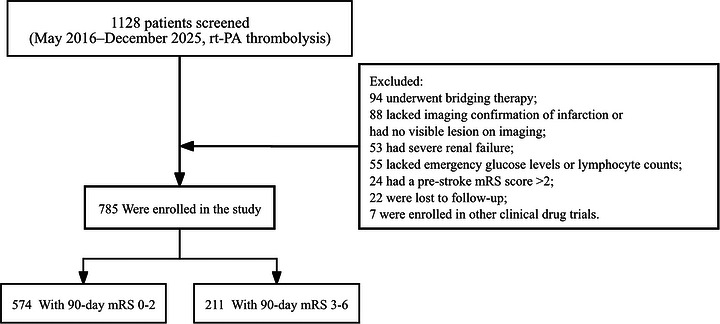
Research flow chart.

A total of 785 patients with AIS treated with IVT were included in the final analysis (Figure 1). Missing data were limited overall, ranging from 0.1% for CRE to 3.8% for Hcy across variables (Figure S1). Poor functional outcome at 90 days occurred in 29.64% of patients. Baseline characteristics according to 90‐day functional outcome are presented in Table 1. Compared with patients with favorable outcome, those with poor outcome were older, had more severe neurological deficits on admission, longer onset‐to‐needle time, and a less favorable vascular risk and laboratory profile.

**TABLE 1:** Baseline characteristics of patients with acute ischemic stroke treated with intravenous thrombolysis according to 90‐day functional outcome.

Variables	Total population (*n* = 785)	Results after 90 days		*p*‐value
		Favorable outcome (mRS ≤ 2, *n* = 574)	Poor outcome (mRS ≥ 3, *n* = 211)	
**Age (years), median (IQR)**	71.00 (63.00, 82.00)	68.00 (61.00, 79.00)	78.00 (69.00, 88.00)	<0.001
**Male, *n* (%)**	516 (65.7)	401 (69.9)	115 (54.5)	<0.001

Risk factors, *n* (%)				
**Smoking**	359 (45.7)	284 (49.5)	75 (35.5)	<0.001
**Diabetes **	273 (34.8)	192 (33.4)	81 (38.4)	0.198
**Stroke/TIA**	181 (23.1)	122 (21.3)	59 (28.0)	0.048
**Hypertension**	566 (72.1)	406 (70.7)	160 (75.8)	0.158
**Coronary artery disease**	151 (19.2)	104 (18.1)	47 (22.3)	0.190
**Atrial fibrillation**	152 (19.4)	86 (15.0)	66 (31.3)	<0.001

Clinical data				
**Time from onset‐to‐needle (min), median (IQR)**	133.00 (93.00, 175.00)	130.00 (90.00, 175.00)	144.00 (105.00, 173.50)	0.010
**sICH, *n* (%)**	43 (5.5)	15 (2.6)	28 (13.3)	<0.001
**Systolic blood pressure (mmHg), median (IQR)**	159.00 (142.00, 174.00)	158.00 (141.00, 171.00)	160.00 (144.00, 180.00)	0.013
**Diastolic blood pressure (mmHg), median (IQR)**	86.00 (78.00, 96.00)	86.00 (78.00, 95.75)	86.00 (76.00, 98.00)	0.657
**Baseline NIHSS, median (IQR)**	4.00 (2.00, 8.00)	3.00 (2.00, 5.00)	8.00 (5.00, 14.00)	<0.001
**END, *n* (%)**	108 (13.8)	38 (6.6)	70 (33.2)	<0.001
**All‐cause mortality at 90 days, *n* (%)**	38 (4.8)	0 (0.0)	38 (18.0)	<0.001
**TOAST classification**				
**Large‐artery atherosclerosis**	296 (37.7)	204 (35.5)	92 (43.6)	<0.001
**Cardioembolic**	154 (19.6)	83 (14.5)	71 (33.6)	
**Small‐vessel occlusion**	282 (35.9)	243 (42.3)	39 (18.5)	
**Other**	53 (6.8)	44 (7.7)	9 (4.3)	
**Admission period (year groups)**				
**2016–2018**	272 (34.6)	198 (34.5)	74 (35.1)	0.768
**2019–2021**	253 (32.2)	189 (32.9)	64 (30.3)	
**2022–2025**	260 (33.1)	187 (32.6)	73 (34.6)	

Laboratory parameters, median (IQR)				
**CRP, mg/L**	5.00 (2.18, 5.00)	5.00 (1.95, 5.00)	5.00 (3.21, 6.54)	0.002
**WBC, 10^9^/L**	7.10 (5.80, 8.50)	7.05 (5.80, 8.50)	7.20 (5.80, 8.55)	0.907
**LYM, 10^9^/L**	1.90 (1.43, 2.48)	1.96 (1.45, 2.57)	1.76 (1.31, 2.13)	<0.001
**RBC, 10^12^/L**	4.63 (4.26, 4.98)	4.67 (4.30, 5.02)	4.54 (4.14, 4.87)	0.002
**PLT, 10^9^/L**	206.00 (171.00, 247.00)	209.00 (172.00, 248.00)	199.00 (169.00, 236.50)	0.124
**Hb, g/L**	142.00 (130.00, 153.00)	143.00 (132.00, 154.00)	137.00 (124.00, 149.00)	<0.001
**HbA1c, %**	6.00 (5.70, 6.70)	6.00 (5.70, 6.60)	6.20 (5.70, 6.95)	0.014
**GLU, mmol/L**	7.29 (6.19, 9.50)	7.11 (6.07, 9.15)	7.95 (6.47, 10.10)	<0.001
**GLR**	4.14 (2.85, 5.88)	3.83 (2.71, 5.66)	4.85 (3.40, 6.88)	<0.001
**TG, mmol/L**	1.17 (0.85, 1.64)	1.21 (0.87, 1.69)	1.10 (0.77, 1.50)	0.008
**TC, mmol/L**	4.64 (3.90, 5.51)	4.70 (3.94, 5.54)	4.46 (3.80, 5.46)	0.165
**HDL‐C, mmol/L**	1.16 (0.96, 1.38)	1.17 (0.98, 1.38)	1.12 (0.94, 1.36)	0.274
**LDL‐C, mmol/L**	2.93 (2.37, 3.60)	2.97 (2.41, 3.61)	2.86 (2.24, 3.46)	0.156
**CRE, umol/L**	78.00 (66.00, 94.00)	77.00 (65.00, 94.00)	79.00 (66.00, 95.00)	0.420
**ALB, g/L**	42.20 (39.20, 44.80)	42.50 (39.82, 45.20)	41.50 (37.95, 43.90)	<0.001
**Hcy, mmol/L**	14.45 (10.92, 19.43)	13.96 (10.54, 19.02)	15.13 (12.05, 21.76)	0.006

Abbreviations: ALB, albumin; CRE, creatinine; CRP, C‐reactive protein; END, early neurological deterioration; GLR, glucose‐to‐lymphocyte ratio; GLU, glucose; Hb, hemoglobin; HbA1c, hemoglobin A1c; Hcy, homocysteine; HDL‐C, high‐density lipoprotein cholesterol; LDL‐C, low‐density lipoprotein cholesterol; LYM, lymphocyte count; NIHSS, National Institutes of Health Stroke Scale; PLT, platelet count; RBC, red blood cell count; sICH, symptomatic intracranial hemorrhage; TC, total cholesterol; TG, triglycerides; TIA, transient ischemic attack; TOAST, Trial of Org 10172 in Acute Stroke Treatment; WBC, white blood cell count.

### 3.2 ROC Analysis of GLR

**FIGURE 2:**
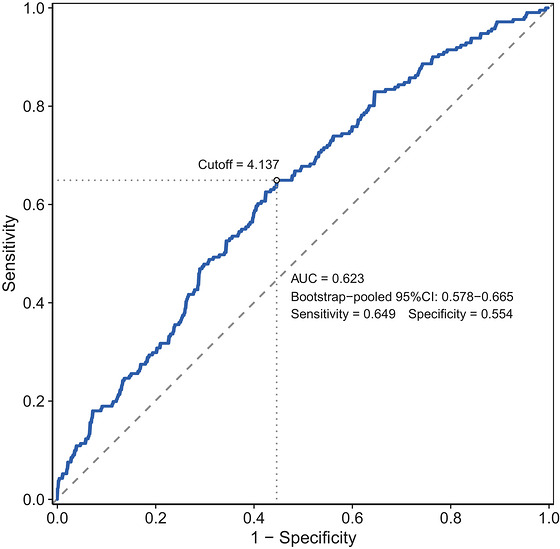
**Receiver operating characteristic curve of GLR for predicting poor functional outcome at 90 days**. *Note*: The ROC curve shows the discriminatory ability of admission GLR for predicting poor functional outcome at 90 days, defined as a modified Rankin Scale score >2. The area under the curve was 0.623, with a bootstrap‐pooled 95% confidence interval of 0.578–0.665. The optimal cutoff value was 4.137, corresponding to a sensitivity of 0.649 and a specificity of 0.554. Estimates were pooled across 20 imputed datasets, with 500 bootstrap resamples per dataset.

ROC analysis indicated that admission GLR had modest discriminatory performance for predicting poor functional outcome at 90 days. The optimal cutoff identified by the Youden index was 4.137. Across the imputed datasets, the pooled AUC was 0.623 (95% CI 0.578–0.665), with pooled sensitivity and specificity of 0.649 and 0.554, respectively (Figure 2).

### 3.3 Association Between GLR and Poor Outcome

In multivariable logistic regression analyses, admission GLR was independently associated with poor 90‐day functional outcome. In the fully adjusted model, continuous GLR was significantly associated with poor outcome (OR = 1.071, 95% CI 1.004–1.144, *p* = 0.038). When analyzed categorically using the ROC‐derived cutoff, patients with high GLR had a significantly increased risk of poor outcome compared with those with low GLR (OR = 1.639, 95% CI 1.057–2.543, *p* = 0.027) (Table 2).

**TABLE 2:** Association of admission glucose‐to‐lymphocyte ratio with poor functional outcome at 90 days in patients with acute ischemic stroke treated with intravenous thrombolysis.

GLR levels	Unadjusted model		Model 1		Model 2		Model 3a		Model 3b	
	OR (95% CI)	*p*‐value	OR (95% CI)	*p*‐value	OR (95% CI)	*p*‐value	OR (95% CI)	*p*‐value	OR (95% CI)	*p*‐value
GLR	1.132 (1.080–1.187)	<0.001	1.074 (1.010–1.143)	0.023	1.071 (1.004–1.144)	0.038	1.071 (1.002–1.144)	0.042	1.080 (1.004–1.162)	0.040
SD‐GLR	1.492 (1.281–1.738)	<0.001	1.259 (1.032–1.536)	0.023	1.249 (1.012–1.541)	0.038	1.246 (1.008–1.541)	0.042	1.282 (1.012–1.624)	0.040

GLR level, low vs. high										
Low	Reference	/	Reference	/	Reference	/	Reference	/	Reference	/
High	2.300 (1.657–3.191)	<0.001	1.680 (1.102–2.560)	0.016	1.639 (1.057–2.543)	0.027	1.657 (1.060–2.592)	0.027	1.775 (1.090–2.889)	0.021

Abbreviations: CI, confidence interval; END, early neurological deterioration; GLR, glucose‐to‐lymphocyte ratio; OR, odds ratio; SD‐GLR, standardized glucose‐to‐lymphocyte ratio per standard deviation increase; sICH, symptomatic intracranial hemorrhage.

*Note*: Poor functional outcome was defined as a modified Rankin Scale score of 3–6 at 90 days. High‐ and low‐GLR groups were classified according to the ROC‐derived cutoff value of 4.137.

Model 1 was adjusted for sex, age, smoking, previous stroke or transient ischemic attack, atrial fibrillation, onset‐to‐needle time, systolic blood pressure, CRP, RBC, Hb, Hcy, TG, and ALB.

Model 2 was further adjusted for study period, sex, age, systolic blood pressure, diastolic blood pressure, onset‐to‐needle time, TOAST classification, previous stroke or transient ischemic attack, atrial fibrillation, diabetes mellitus, hypertension, coronary artery disease, smoking, baseline NIHSS score, HbA1c, CRP, WBC, RBC, Hb, PLT, CRE, Hcy, TG, HDL‐C, LDL‐C, and ALB.

Model 3a was based on Model 2 with additional adjustment for sICH.

Model 3b was based on Model 2 with additional adjustment for END.

### 3.4 Robustness and Sensitivity Analyses

Several additional analyses were performed to assess the robustness of the main findings. Sensitivity models additionally adjusting for symptomatic intracranial hemorrhage (sICH) or early neurological deterioration (END) yielded broadly similar estimates (Table 2). To address potential collinearity among lipid variables, an alternative lipid‐adjusted model retaining total cholesterol (CHOL) while excluding low‐density lipoprotein cholesterol (LDL‐C) was fitted, and the association between continuous GLR and poor outcome remained materially unchanged (OR = 1.071, 95% CI 1.003–1.143, *p* = 0.040).

Covariate balance between the low‐ and high‐GLR groups improved substantially after weighting and was further improved after truncation of extreme weights. Most covariates achieved adequate balance, whereas WBC showed slight residual imbalance after stabilized weighting and approached the conventional threshold after truncation (Table S1 and Figure S2). IPW analyses based on the dichotomized GLR variable further supported the main findings. Across three weighted models with progressive residual adjustment, the ORs for poor outcome ranged from 1.653 to 1.666, and all associations remained statistically significant (Table 3).

**TABLE 3:** Association of high versus low glucose‐to‐lymphocyte ratio with poor functional outcome at 90 days under inverse probability weighting and truncated weighting.

**GLR levels**		**Model 1**		**Model 2**		**Model 3**	
		**OR (95% CI)**	***p*‐value**	**OR (95% CI)**	***p*‐value**	**OR (95% CI)**	***p*‐value**
IPW	Low	Reference	/	Reference	/	Reference	/
	High	1.666 (1.097–2.529)	0.017	1.658 (1.073–2.561)	0.023	1.653 (1.062–2.573)	0.026
Trunc	Low	Reference	/	Reference	/	Reference	/
	High	1.706 (1.124–2.589)	0.012	1.694 (1.096–2.618)	0.018	1.689 (1.085–2.630)	0.020

Abbreviations: IPW, inverse probability weighting; Trunc, truncated weighting.

*Note*: Poor functional outcome was defined as a modified Rankin Scale score of 3–6 at 90 days. High‐ and low‐GLR groups were defined according to the ROC‐derived cutoff value of 4.137. Stabilized IPW weights for the average treatment effect were estimated using propensity scores derived from baseline covariates. Truncated weights were obtained by winsorizing stabilized IPW weights at the first and 99th percentiles.

Model 1 was additionally adjusted for core covariates (age, baseline NIHSS score, and WBC).

Model 2 was further adjusted for the five covariates with the largest residual absolute SMDs after weighting (HbA1c, PLT, CRP, Hcy, and ALB).

Model 3 was further adjusted for the next five covariates with the largest residual absolute SMDs after weighting (TG, systolic blood pressure, TOAST classification, LDL‐C, and Hb).

In quartile analyses, only the third quartile showed a borderline association with poor outcome compared with the lowest quartile (OR = 1.858, 95% CI 1.001–3.451, *p* = 0.050), whereas the second and fourth quartiles were not statistically significant; no significant linear trend across quartiles was observed (*p* for trend = 0.262) (Table S2). In sensitivity analyses using mRS 0–1 versus 2–6 as the alternative outcome definition, neither continuous GLR (OR = 1.014, 95% CI 0.954–1.078, *p* = 0.655) nor categorical GLR (OR = 1.258, 95% CI 0.863–1.834, *p* = 0.233) was significantly associated with outcome (Table S3).

### 3.5 Shape and Subgroup Analyses

**FIGURE 3:**
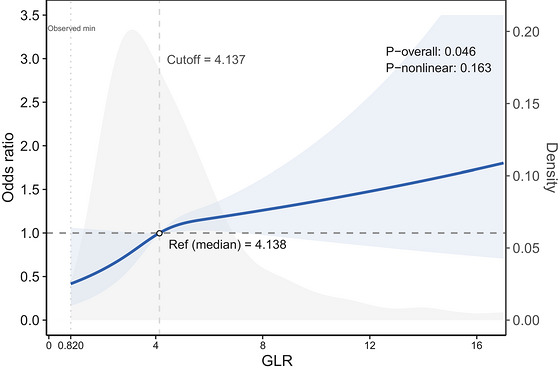
**Restricted cubic spline analysis of the association between GLR and poor functional outcome**. *Note*: Odds ratios were derived from the fully adjusted logistic regression model. The solid line represents the adjusted odds ratio for poor functional outcome across the distribution of GLR, and the shaded area indicates the 95% confidence interval. The median GLR value (4.138) was used as the reference. The vertical dashed line indicates the ROC‐derived cutoff value of 4.137. The distribution of GLR is shown by the density curve along the *x*‐axis. The overall association was statistically significant (*p*‐overall = 0.046), whereas no significant evidence of nonlinearity was observed (*p*‐nonlinear = 0.163).

**FIGURE 4:**
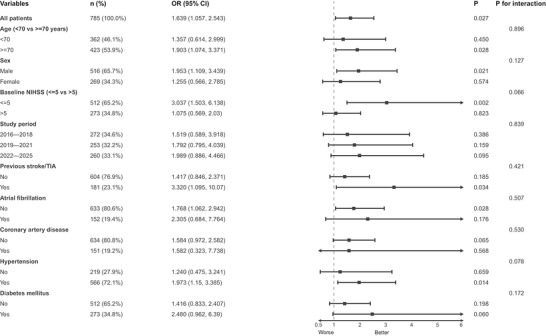
**Subgroup analysis of the association between dichotomized GLR and poor functional outcome**. *Note*: Forest plot showing the odds ratios and 95% confidence intervals for poor functional outcome associated with high versus low GLR in the overall population and across prespecified subgroups, including age, sex, baseline NIHSS, study period, previous stroke, atrial fibrillation, coronary artery disease, hypertension, and diabetes mellitus. *p* values for interaction were calculated to assess heterogeneity across subgroups.

Restricted cubic spline analysis showed an overall association between GLR and poor outcome (*p* for overall association = 0.046), without significant evidence of nonlinearity (*p* for nonlinearity = 0.163), indicating a broadly linear relationship across the observed GLR range (Figure 3). Prespecified subgroup analyses are presented in Figures 4, S3, and S4. The direction of association between higher GLR and poor outcome was generally consistent across clinically relevant subgroups, and no statistically significant interaction was detected.

### 3.6 Incremental Predictive Performance and Internal Validation

Addition of GLR to the fully adjusted model resulted in only a modest improvement in predictive performance. The AUC increased from 0.836 to 0.839, and this difference was not statistically significant by the DeLong test (*p* = 0.262). However, likelihood ratio testing indicated improved model fit (*p* = 0.037). The continuous net reclassification improvement and integrated discrimination improvement were 0.195 and 0.004, respectively.

Bootstrap internal validation yielded an apparent AUC of 0.839 and an optimism‐corrected AUC of 0.803. The Brier score of the extended model was 0.134. The apparent calibration of the extended model is shown in Figure S5.

### 3.7 Collinearity and Correlation Diagnostics

**FIGURE 5:**
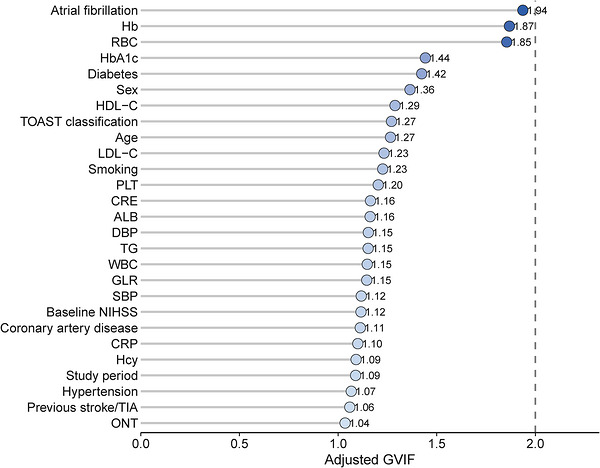
**Collinearity diagnostics for Model 2**. *Note*: Adjusted generalized variance inflation factors are shown for variables included in Model 2. To improve comparability across variables with different degrees of freedom, collinearity was summarized as GVIF^1/(2×Df)^. The observed values indicated acceptable levels of multicollinearity in the model.

Collinearity diagnostics suggested acceptable levels of multicollinearity in the main adjusted model (Figure 5). Correlation analyses among continuous covariates demonstrated expected interrelationships among several biologically related variables but did not materially affect the robustness of the association between GLR and poor outcome (Figure S6).

## 4 Discussion

In this retrospective single‐center study of patients with acute ischemic stroke treated with intravenous thrombolysis, we found that a higher admission glucose‐to‐lymphocyte ratio was independently associated with an increased risk of poor 90‐day functional outcome. This association remained robust across multiple analytic approaches, including multivariable adjustment, additional sensitivity analyses, and inverse probability weighting. Restricted cubic spline analysis further suggested that the relationship between GLR and poor outcome was broadly linear across the observed range, while prespecified subgroup analyses showed a generally consistent direction of association across clinically relevant subgroups. However, the discriminatory performance of GLR alone was modest, and the association was attenuated in sensitivity analyses using an alternative dichotomization of mRS.

A plausible biological explanation for these findings is that GLR integrates two pathophysiological domains that are highly relevant to stroke prognosis: metabolic stress and immune dysregulation. Admission hyperglycemia is common after AIS and has been consistently associated with larger infarct size, blood–brain barrier disruption, oxidative stress, endothelial dysfunction, and worse neurological recovery (Capes et al. 2001; Kamada et al. 2007; Kruyt et al. 2010; Luitse et al. 2012). Hyperglycemia may also aggravate ischemia reperfusion injury by promoting leukocyte adhesion, microvascular dysfunction, inflammatory activation, and cerebral edema (Bruno et al. 2002). In thrombolysis treated patients in particular, elevated glucose has been linked to a higher risk of hemorrhagic transformation and poorer functional outcomes (Demchuk et al. 1999; Poppe et al. 2009; Westendorp et al. 2011). These observations support the concept that acute glycemic disturbance is not merely an epiphenomenon of stroke severity, but may also participate directly in secondary brain injury.

At the same time, lymphocyte count may reflect an important component of the host response after stroke. Stroke‐associated immune alterations, often characterized by lymphopenia, are thought to result from activation of the sympathetic nervous system and the hypothalamic‐pituitary‐adrenal axis, leading to immunosuppression and increased susceptibility to infection (Chamorro et al. 2012; Prass et al. 2003; Urra et al. 2009; Zhang et al. 2017). Lower lymphocyte counts have been associated with stroke associated pneumonia, sepsis, in hospital complications, and worse long term outcomes (Smith et al. 2004; Zhou et al. 2025). Beyond anti‐infective defense, lymphocytes are also involved in regulating inflammatory balance and tissue repair after cerebral ischemia. Accordingly, a biomarker that combines blood glucose and lymphocyte count may better capture the combined impact of acute metabolic disturbance and post‐stroke immune dysfunction than either parameter alone.

Our findings are generally consistent with the growing literature supporting GLR as a prognostic biomarker across multiple clinical settings. Previous studies have suggested that GLR has prognostic relevance in cardiovascular disease, diabetes‐related populations, and stroke‐related complications (Lei et al. 2025; Liu and Hu 2023; Lyu et al. 2025; Serhatlioglu et al. 2024; Wu et al. 2025; Zhou et al. 2025). For example, higher GLR has been linked to increased mortality in heart failure and to the occurrence of stroke‐associated pneumonia in acute stroke cohorts (Wu et al. 2025; Zhou et al. 2025). In the present study, we extend this line of evidence by focusing specifically on AIS patients undergoing IVT and by examining 90‐day functional outcome. This is clinically relevant because patients treated with thrombolysis represent a distinct population in whom early risk stratification may influence monitoring intensity, complication prevention, and post‐acute management.

Several aspects of our findings warrant careful interpretation. Although continuous GLR was significantly associated with poor outcome, the effect size was modest and its discriminatory performance was limited when used alone, suggesting that GLR is unlikely to serve as a standalone prognostic marker. Rather, it may be more useful as an inexpensive and readily available component of a broader risk assessment framework. In this sense, GLR may be better interpreted as an associated biomarker reflecting metabolic and immune‐inflammatory stress than as a strong individual predictor for clinical decision‐making. In addition, adding GLR to the fully adjusted model resulted in only a small and non‐significant increase in AUC by the DeLong test, whereas the likelihood ratio test suggested improved overall model fit. Together, these findings indicate that GLR may provide incremental prognostic information despite limited gains in discrimination. Restricted cubic spline analysis further supported a broadly linear association across the observed range, although the quartile‐based analysis suggested some instability across categorical groupings and did not support a significant linear trend, arguing against overinterpretation of threshold effects. Moreover, the non‐significant result in the sensitivity analysis using an alternative dichotomization of the modified Rankin Scale indicates that the prognostic relevance of GLR may depend partly on outcome definition.

Subgroup analyses supported the robustness of the main finding, with generally consistent directions of association across prespecified subgroups and no strong evidence of interaction. However, the absence of statistically significant interaction should not be taken as definitive evidence of homogeneity, given the limited power of subgroup analyses in observational studies to detect moderate effect modification. From a clinical perspective, GLR also has several practical advantages. It is derived from routine admission laboratory tests, requires no additional cost or specialized equipment, and can be calculated rapidly in the acute setting. These features make it a pragmatic biomarker for early bedside risk stratification. Nevertheless, because GLR is influenced by multiple factors, including stress hyperglycemia, pre‐existing diabetes, acute infection, corticosteroid exposure, and other comorbid conditions, it should be interpreted within the broader clinical context rather than used in isolation.

Several limitations of this study should also be acknowledged. The retrospective single‐center design may have introduced selection bias and limited the generalizability of our findings. Moreover, as an observational retrospective study, our results demonstrate association rather than causation, and elevated GLR may partly reflect stroke severity, systemic stress responses, or other unmeasured clinical factors rather than a direct pathophysiological effect. Temporal changes in stroke care over the long study period may also have affected patient characteristics and outcomes, and residual temporal heterogeneity cannot be fully excluded despite adjustment for calendar time. Although we applied multiple imputation, multivariable adjustment, and inverse probability weighting, residual confounding from unmeasured factors remains possible. In addition, the predictive gain associated with GLR was modest, admission GLR was the only measure assessed, and some secondary analyses were not statistically significant. External validation in independent multicenter cohorts is therefore required.

In conclusion, admission GLR was independently associated with poor 90‐day functional outcome in patients with acute ischemic stroke undergoing intravenous thrombolysis. As a simple composite biomarker integrating metabolic and immune‐inflammatory information, GLR may offer useful supplementary prognostic information in acute stroke. However, its standalone discriminatory performance and incremental prognostic value were modest, and further studies are needed to validate its clinical utility.

## Author Contributions

**Dong Zhang**: Conceptualization, methodology, software, data curation, investigation, validation, formal analysis, visualization, writing – original draft. **Ying Zong**: Methodology, software, investigation, validation, writing – original draft, visualization. **Xiaoyan Qin**: Software, investigation, validation, formal analysis, visualization, writing – original draft. **Ruinan Ma**: Data curation, investigation, formal analysis. **Zhizhang Li**: Software, data curation, formal analysis. **Xiaoguang Zhang**: Data curation, investigation. **Ying Ding**: Data curation, investigation. **Yueying Bai**: Software, data curation, investigation. **Liang Hu**: Supervision, funding acquisition, project administration, resources, writing – review & editing. **Yunhua Yue**: Supervision, funding acquisition, project administration, resources, writing – review & editing. All authors have read and approved the final manuscript.

## Funding

This study received financial support from a project grant provided by the Yangpu District Health Commission of Shanghai (Project No: YPM202305).

## Ethics Statement

The ethics committee at Yangpu Hospital, Tongji University School of Medicine, approved the study (LL‐2021‐LW‐003) on February 22, 2021. All procedures involving human participants adhered to the ethical standards of the Yangpu Hospital Ethics Committee at Tongji University School of Medicine, as well as the 1964 Helsinki Declaration and its subsequent amendments or comparable standards.

## Conflicts of Interest

The authors declare that they have no conflicts of interest.

## Supporting information




**Figure S1. Proportion of missing data across variables**. *Note*: Bar plot showing the percentage of missing values for variables with incomplete data in the study cohort. Overall, missingness was low across variables, supporting the use of multiple imputation for handling missing covariate data.

**Figure S2. Propensity score distribution before and after weighting**. *Note*: Density plots of propensity scores for the low‐ and high‐GLR groups before and after inverse probability weighting with stabilized average treatment effect weights. The increased overlap in propensity score distributions after weighting suggests improved balance between groups.

**Figure S3. Subgroup analysis of the association between continuous GLR per 1‐unit increase and poor functional outcome**. *Note*: Forest plot showing the odds ratios and 95% confidence intervals for poor functional outcome associated with each 1‐unit increase in GLR in the overall population and across prespecified subgroups, including age, sex, baseline NIHSS, study period, previous stroke, atrial fibrillation, coronary artery disease, hypertension, and diabetes mellitus. *p* values for interaction were calculated to assess heterogeneity across subgroups.

**Figure S4. Subgroup analysis of the association between continuous GLR per SD increase and poor functional outcome**. *Note*: Forest plot showing the odds ratios and 95% confidence intervals for poor functional outcome associated with each standard deviation increase in GLR in the overall population and across prespecified subgroups, including age, sex, baseline NIHSS, study period, previous stroke, atrial fibrillation, coronary artery disease, hypertension, and diabetes mellitus. *p* values for interaction were calculated to assess heterogeneity across subgroups.

**Figure S5. Apparent calibration plot of the extended model**. *Note*: Apparent calibration was assessed by comparing mean predicted probabilities with observed event rates across the range of predicted risk for the extended model. Closer agreement between predicted and observed values indicates better calibration.

**Figure S6. Correlation heatmap of continuous covariates in main Model 2**. *Note*: The heatmap shows pairwise correlation coefficients among continuous covariates included in main Model 2. Color intensity indicates the direction and strength of correlation between variables and complements the collinearity diagnostics.

**Table S1. Covariate balance between low‐ and high‐GLR groups before weighting, after stabilized inverse probability weighting, and after truncated weighting**. *Note*: After inverse probability weighting, the displayed counts represent weighted sample sizes and may therefore be non‐integer values. Covariate balance was primarily assessed using standardized mean differences (SMDs). High‐ and low‐GLR groups were defined according to the ROC‐derived cutoff value of 4.137. Continuous variables are presented as median (interquartile range), and categorical variables as number (percentage). An SMD of less than 0.1 was considered indicative of adequate balance. Abbreviations: GLR, glucose‐to‐lymphocyte ratio; IPW, inverse probability weighting; SMD, standardized mean difference.
**Table S2. Logistic regression analysis of quartiles of glucose‐to‐lymphocyte ratio and poor functional outcome**. *Note*: Poor functional outcome was defined as a modified Rankin Scale score of 3–6 at 90 days. GLR was categorized into quartiles, with the lowest quartile (Q1) as the reference group. *p* for trend was calculated by assigning the median GLR value of each quartile to all participants in that quartile and entering this variable as a continuous term in the adjusted logistic regression model. Odds ratios were estimated using logistic regression with the same covariate adjustment set as the main Model 2, including study period, sex, age, systolic blood pressure, diastolic blood pressure, onset‐to‐needle time, TOAST classification, previous stroke or transient ischemic attack, atrial fibrillation, diabetes mellitus, hypertension, coronary artery disease, smoking, baseline NIHSS score, HbA1c, CRP, WBC, RBC, Hb, PLT, CRE, Hcy, TG, HDL‐C, LDL‐C, and ALB.
**Table S3. Sensitivity analysis using an alternative dichotomization of 90‐day functional outcome**. *Note*: In this sensitivity analysis, poor functional outcome was redefined as an mRS score of 2–6 at 90 days, and favorable outcome as an mRS score of 0–1. High‐ and low‐GLR groups were defined according to the ROC‐derived cutoff value of 4.137. Both analyses used the same covariate adjustment set as the main Model 2, including study period, sex, age, systolic blood pressure, diastolic blood pressure, onset‐to‐needle time, TOAST classification, previous stroke or transient ischemic attack, atrial fibrillation, diabetes mellitus, hypertension, coronary artery disease, smoking, baseline NIHSS score, HbA1c, CRP, WBC, RBC, Hb, PLT, CRE, Hcy, TG, HDL‐C, LDL‐C, and ALB.

## Data Availability

The data that support the findings of this study are available from the corresponding author upon reasonable request.
