# Supplementary figures and images for: Association of Admission Glucose‐to‐Lymphocyte Ratio With 90‐Day Functional Outcome in Patients With Acute Ischemic Stroke Treated With Intravenous Thrombolysis
Source: Brain Behav. 2026 May 11;16(5):e71484. doi: 10.1002/brb3.71484 (PMC13159543; doi:10.1002/brb3.71484)

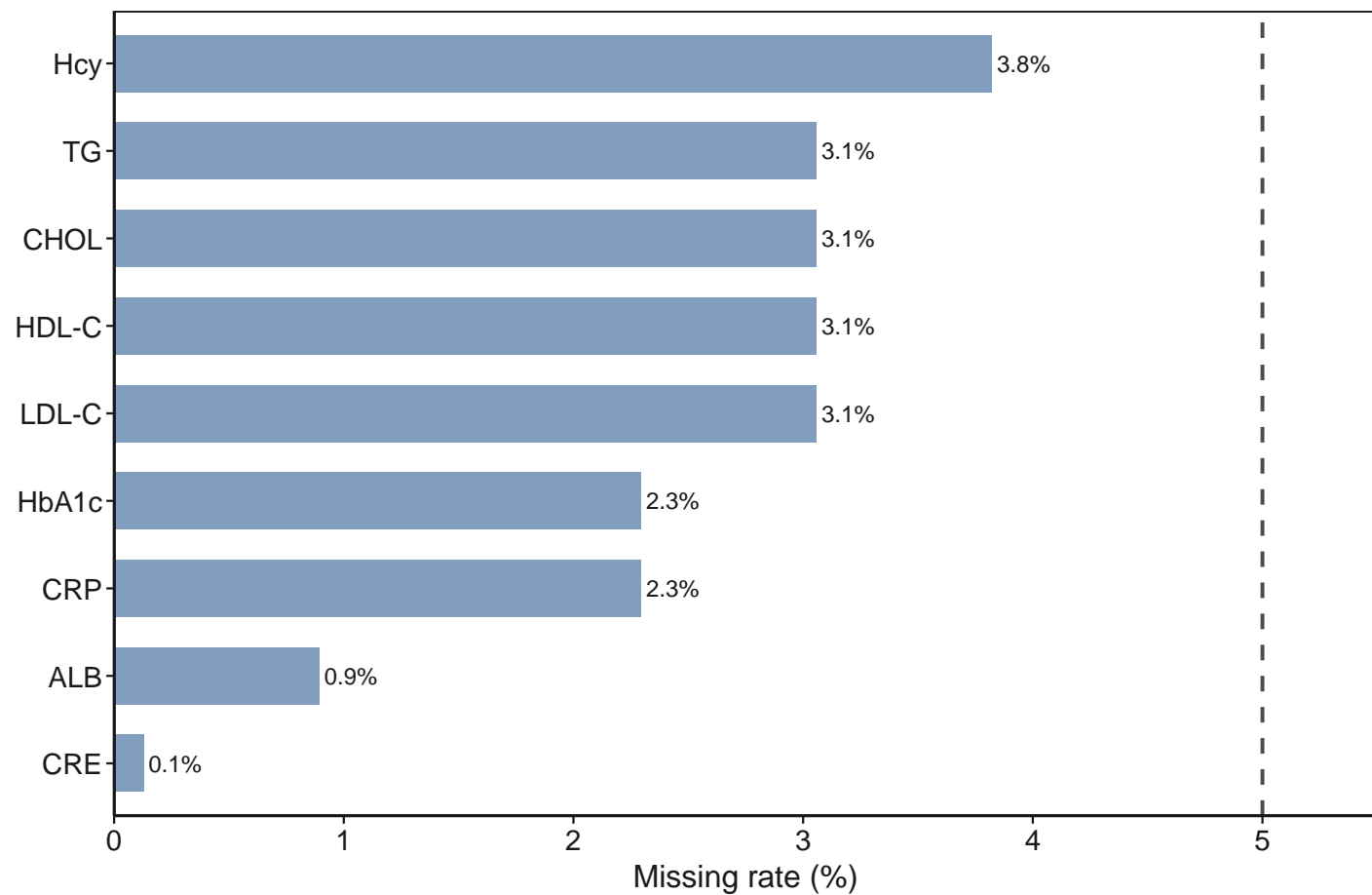

Supplement: Supplementary file 1 — Figure S1. Proportion of missing data across variables. Note: Bar plot showing the percentage of missing values for variables with incomplete data in the study cohort. Overall, missingness was low across variables, supporting the use of multiple imputation for handling missing covariate data. [file BRB3-16-e71484-s005.pdf]

# Propensity Score Distribution Before and After Weighting

Stabilized ATE weights

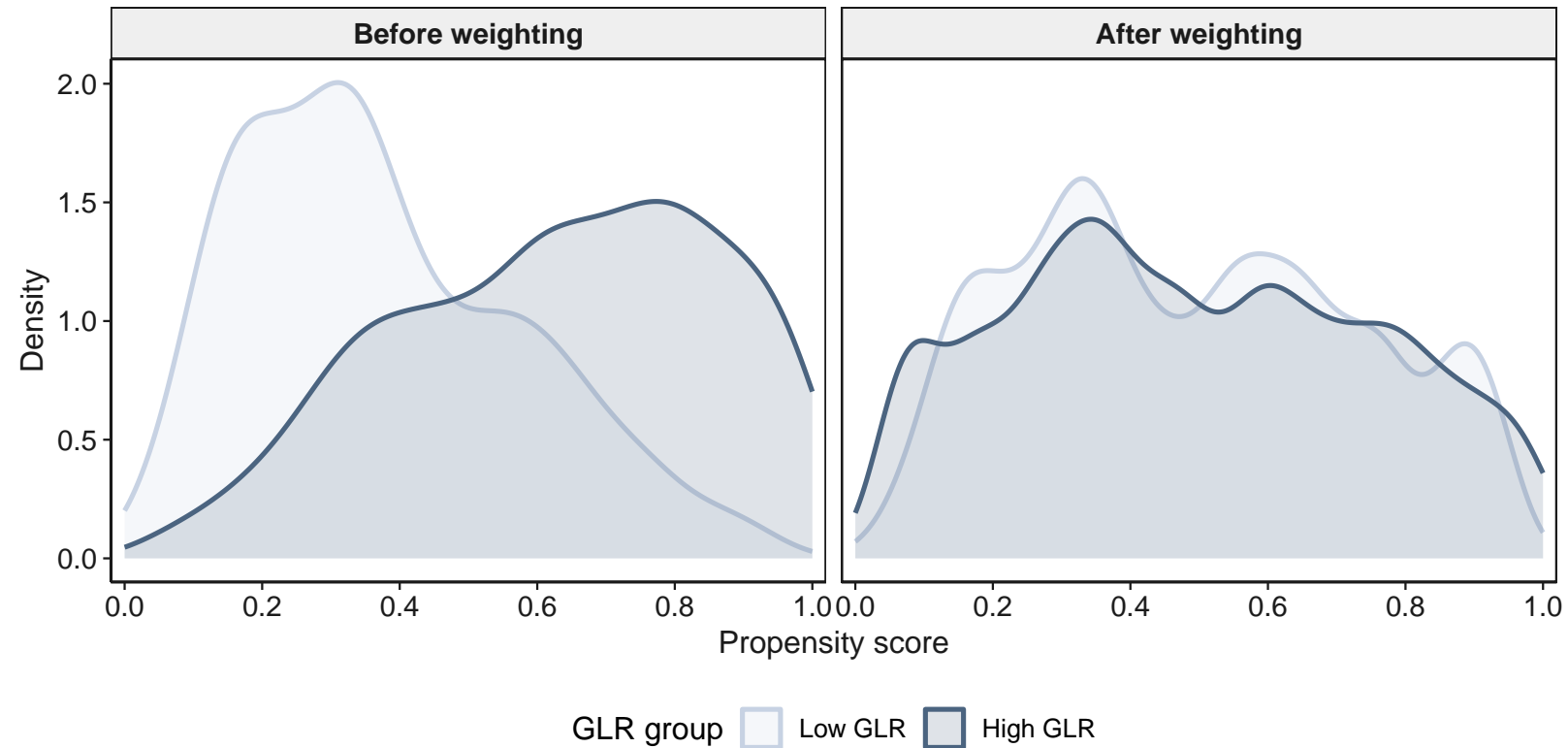

Supplement: Supplementary file 2 — Figure S2. Propensity score distribution before and after weighting. Note: Density plots of propensity scores for the low‐ and high‐GLR groups before and after inverse probability weighting with stabilized average treatment effect weights. The increased overlap in propensity score distributions after weighting suggests improved balance between groups. [file BRB3-16-e71484-s001.pdf]

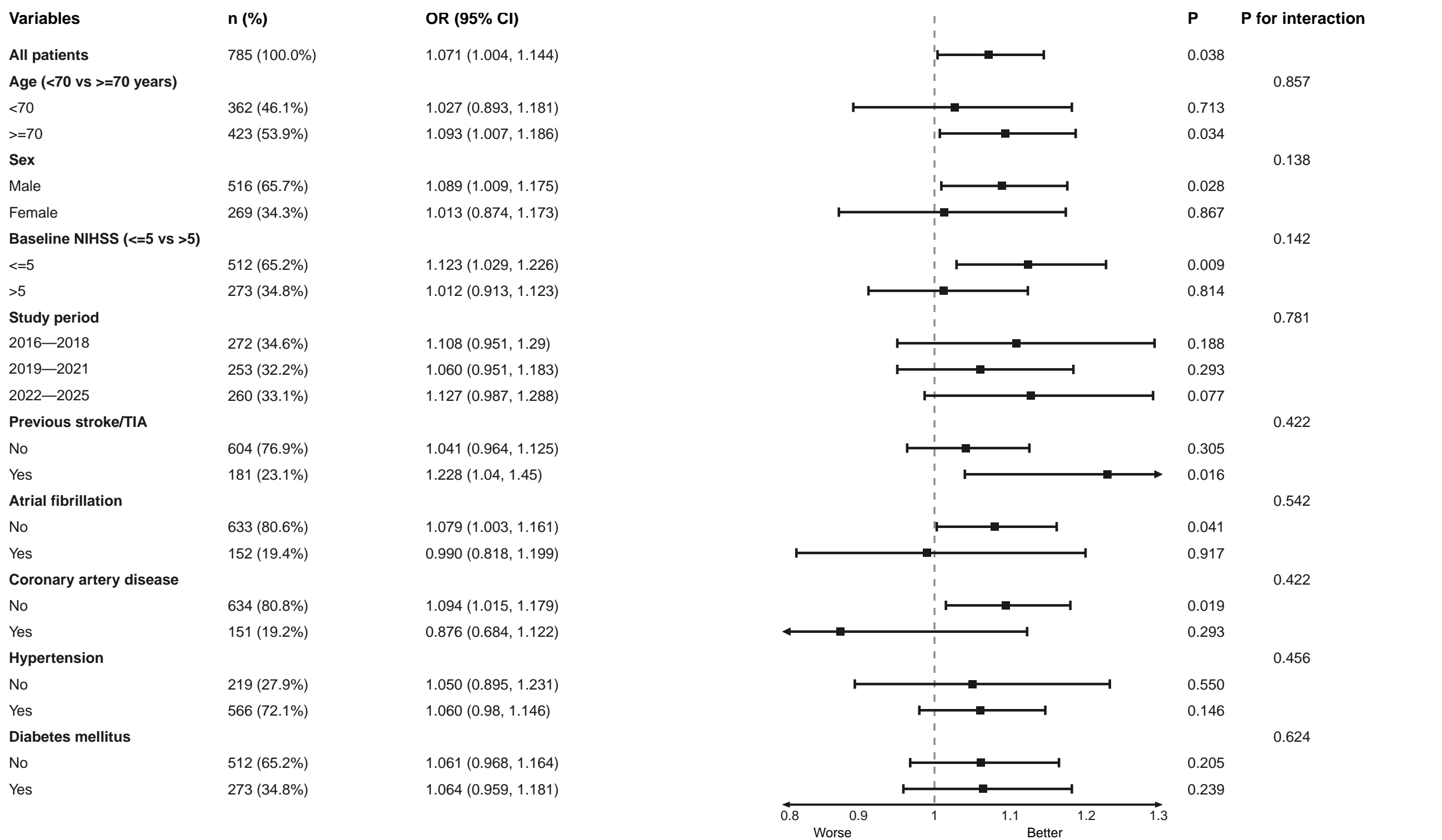

Supplement: Supplementary file 3 — Figure S3. Subgroup analysis of the association between continuous GLR per 1‐unit increase and poor functional outcome. Note: Forest plot showing the odds ratios and 95% confidence intervals for poor functional outcome associated with each 1‐unit increase in GLR in the overall population and across prespecified subgroups, including age, sex, baseline NIHSS, study period, previous stroke, atrial fibrillation, coronary artery disease, hypertension, and diabetes mellitus. p values for interaction were calculated to assess heterogeneity across subgroups. [file BRB3-16-e71484-s006.pdf]

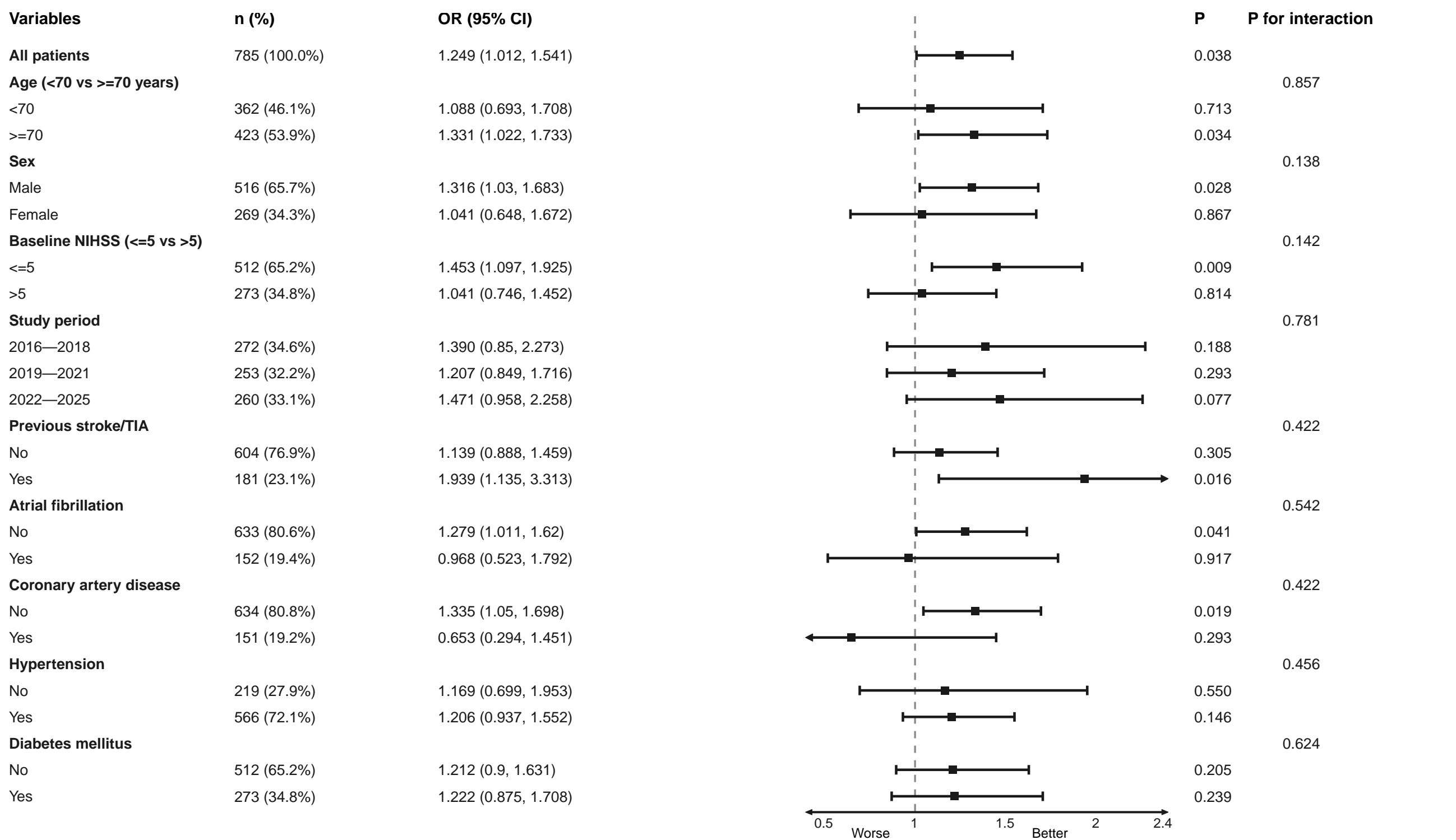

Supplement: Supplementary file 4 — Figure S4. Subgroup analysis of the association between continuous GLR per SD increase and poor functional outcome. Note: Forest plot showing the odds ratios and 95% confidence intervals for poor functional outcome associated with each standard deviation increase in GLR in the overall population and across prespecified subgroups, including age, sex, baseline NIHSS, study period, previous stroke, atrial fibrillation, coronary artery disease, hypertension, and diabetes mellitus. p values for interaction were calculated to assess heterogeneity across subgroups. [file BRB3-16-e71484-s004.pdf]

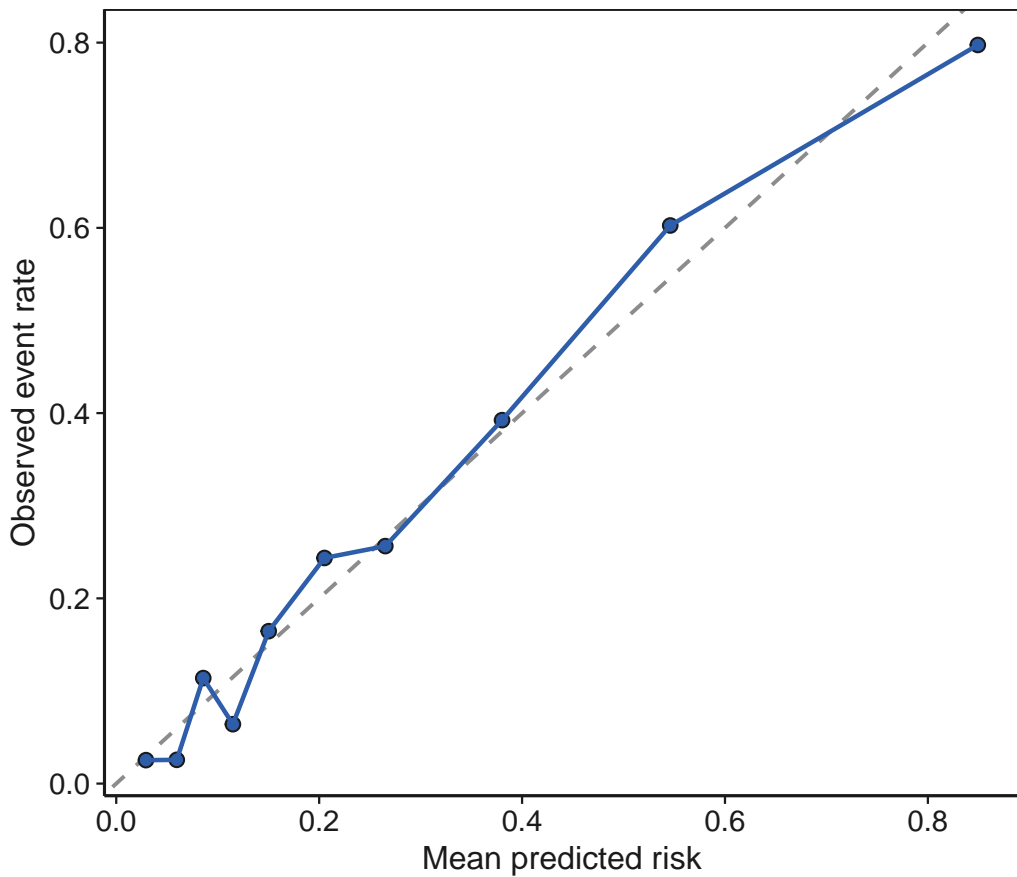

Supplement: Supplementary file 5 — Figure S5. Apparent calibration plot of the extended model. Note: Apparent calibration was assessed by comparing mean predicted probabilities with observed event rates across the range of predicted risk for the extended model. Closer agreement between predicted and observed values indicates better calibration. [file BRB3-16-e71484-s003.pdf]

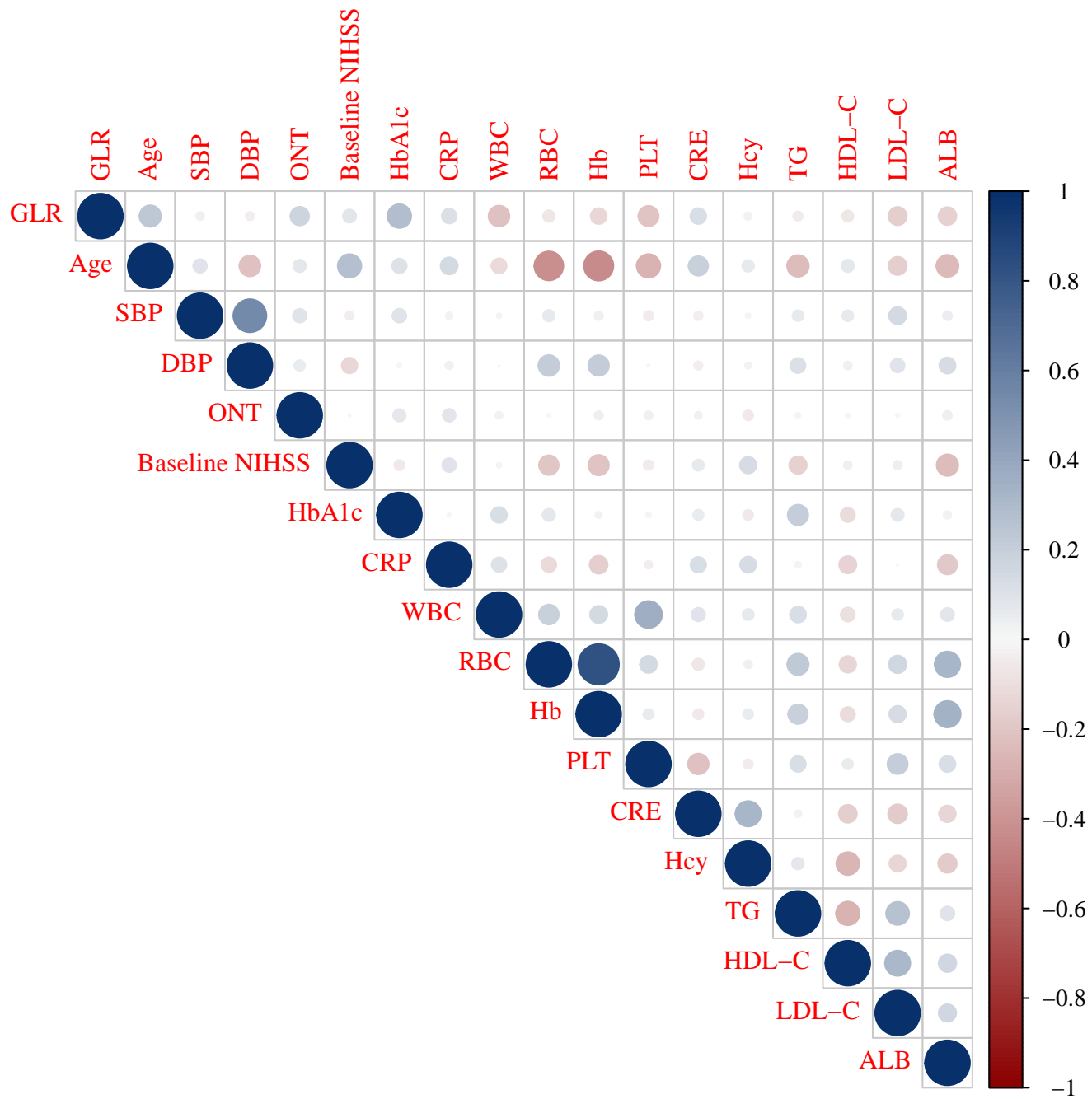

Supplement: Supplementary file 6 — Figure S6. Correlation heatmap of continuous covariates in main Model 2. Note: The heatmap shows pairwise correlation coefficients among continuous covariates included in main Model 2. Color intensity indicates the direction and strength of correlation between variables and complements the collinearity diagnostics. [file BRB3-16-e71484-s002.pdf]
