# Supplementary material for: Association of Admission Glucose‐to‐Lymphocyte Ratio With 90‐Day Functional Outcome in Patients With Acute Ischemic Stroke Treated With Intravenous Thrombolysis
Source: Brain Behav. 2026 May 11;16(5):e71484. doi: 10.1002/brb3.71484 (PMC13159543; doi:10.1002/brb3.71484)
Supplement: Supplementary file 7 — Table S1. Covariate balance between low‐ and high‐GLR groups before weighting, after stabilized inverse probability weighting, and after truncated weighting. Note: After inverse probability weighting, the displayed counts represent weighted sample sizes and may therefore be non‐integer values. Covariate balance was primarily assessed using standardized mean differences (SMDs). High‐ and low‐GLR groups were defined according to the ROC‐derived cutoff value of 4.137. Continuous variables are presented as median (interquartile range), and categorical variables as number (percentage). An SMD of less than 0.1 was considered indicative of adequate balance. Abbreviations: GLR, glucose‐to‐lymphocyte ratio; IPW, inverse probability weighting; SMD, standardized mean difference. Table S2. Logistic regression analysis of quartiles of glucose‐to‐lymphocyte ratio and poor functional outcome. Note: Poor functional outcome was defined as a modified Rankin Scale score of 3–6 at 90 days. GLR was categorized into quartiles, with the lowest quartile (Q1) as the reference group. p for trend was calculated by assigning the median GLR value of each quartile to all participants in that quartile and entering this variable as a continuous term in the adjusted logistic regression model. Odds ratios were estimated using logistic regression with the same covariate adjustment set as the main Model 2, including study period, sex, age, systolic blood pressure, diastolic blood pressure, onset‐to‐needle time, TOAST classification, previous stroke or transient ischemic attack, atrial fibrillation, diabetes mellitus, hypertension, coronary artery disease, smoking, baseline NIHSS score, HbA1c, CRP, WBC, RBC, Hb, PLT, CRE, Hcy, TG, HDL‐C, LDL‐C, and ALB. Table S3. Sensitivity analysis using an alternative dichotomization of 90‐day functional outcome. Note: In this sensitivity analysis, poor functional outcome was redefined as an mRS score of 2–6 at 90 days, and favorable outcome as an mRS score of 0–1. [file BRB3-16-e71484-s007.docx]

**Supplementary Table 1. Covariate Balance Between Low and High GLR Groups Before Weighting, After Stabilized Inverse Probability Weighting, and After Truncated Weighting.**

| **Variables** | **Before weighting** | | | | **After stabilized IPW** | | | | **After truncated weighting** | | | |
| --- | --- | --- | --- | --- | --- | --- | --- | --- | --- | --- | --- | --- |
|  | **Low GLR** | **High GLR** | **SMD** | **P-value** | **Low GLR** | **High GLR** | **SMD** | **P-value** | **Low GLR** | **High GLR** | **SMD** | **P-value** |
| **Age(years), median (IQR)** | 68 (61, 78) | 74 (66, 84) | 0.386 | <0.001 | 71 (62, 84) | 71 (63, 83) | -0.031 | 0.716 | 70 (62, 83) | 72 (63, 83) | 0.016 | 0.435 |
| **Male, n(%)** | 252.0 (64.3%) | 264.0 (67.2%) | 0.029 | 0.394 | 252.8 (66.1%) | 269.0 (67%) | 0.009 | 0.834 | 129.4 (34.4%) | 130.2 (33.4%) | 0.011 | 0.791 |
| **Risk factors, n(%)** | | | | | | | | | | | | |
| **Smoking** | 193.0 (49.2%) | 166.0 (42.2%) | -0.07 | 0.049 | 172.5 (45.1%) | 190.6 (47.5%) | 0.024 | 0.602 | 169.7 (45.2%) | 181.4 (46.5%) | 0.013 | 0.750 |
| **Diabetes** | 81.0 (20.7%) | 192.0 (48.9%) | 0.282 | <0.001 | 133.7 (35%) | 136.5 (34%) | -0.01 | 0.822 | 127.3 (33.9%) | 136.5 (35%) | 0.011 | 0.787 |
| **Stroke/TIA** | 64.0 (16.3%) | 117.0 (29.8%) | 0.134 | <0.001 | 84.1 (22%) | 94.3 (23.5%) | 0.015 | 0.701 | 81.4 (21.7%) | 92.5 (23.7%) | 0.021 | 0.567 |
| **Hypertension** | 267.0 (68.1%) | 299.0 (76.1%) | 0.080 | 0.013 | 273.5 (71.6%) | 284.8 (70.9%) | -0.006 | 0.881 | 267.1 (71.1%) | 278 (71.3%) | 0.002 | 0.956 |
| **Coronary artery disease** | 62.0 (15.8%) | 89.0 (22.6%) | 0.068 | 0.015 | 72.4 (18.9%) | 72.0 (17.9%) | -0.010 | 0.764 | 69.6 (18.5%) | 72 (18.5%) | -0.001 | 0.979 |
| **Atrial fibrillation** | 60.0 (15.3%) | 92.0 (23.4%) | 0.081 | 0.004 | 72.8 (19%) | 83.0 (20.7%) | 0.016 | 0.647 | 71.8 (19.1%) | 80.8 (20.7%) | 0.016 | 0.636 |
| **Clinical data** | | | | | | | | | | | | |
| **Time from onset-to-needle(min), median (IQR)** | 121.00 (86.75, 170.00) | 144.00 (105.00, 180.00) | 0.262 | <0.001 | 129.00 (92.00, 182.00) | 135.00 (99.00, 175.00) | -0.013 | 0.767 | 127.00 (92.00, 178.00) | 136.00 (100.00, 175.00) | 0.028 | 0.447 |
| **Systolic blood pressure(mmHg), median (IQR)** | 158.50 (142.00, 172.25) | 159.00 (142.00, 175.00) | -0.043 | 0.595 | 159.00 (141.00, 173.00) | 156.00 (142.00, 175.00) | -0.034 | 0.920 | 159.00 (142.00, 173.00) | 157.00 (143.00, 175.00) | -0.015 | 0.801 |
| **Diastolic blood pressure(mmHg), median (IQR)** | 87.00 (78.00, 98.00) | 86.00 (76.00, 95.00) | -0.090 | 0.144 | 87.00 (77.00, 97.00) | 86.00 (77.00, 96.00) | -0.002 | 0.789 | 87.00 (77.00, 96.00) | 86.00 (78.00, 96.00) | 0.005 | 0.859 |
| **Baseline NIHSS, median (IQR)** | 4 (2, 7) | 4 (3, 8) | 0.162 | 0.021 | 4 (2, 8) | 4 (3, 8) | 0.017 | 0.380 | 4 (2, 8) | 4 (3, 8) | 0.026 | 0.398 |
| **TOAST classification, n (%)** | | | | | | | | | | | | |
| **Large-artery atherosclerosis** | 138.0 (35.2%) | 158.0 (40.2%) | 0.123 | 0.002 | 146.6 (38.4%) | 155 (38.6%) | 0.031 | 0.851 | 141.2 (37.6%) | 150.8 (38.7%) | 0.032 | 0.876 |
| **Cardioembolic** | 63.0 (16.1%) | 91.0 (23.2%) |  |  | 73.1 (19.1%) | 82.7 (20.6%) |  |  | 72.1 (19.2%) | 80.4 (20.6%) |  |  |
| **Small-vessel occlusion** | 165.0 (42.1%) | 117.0 (29.8%) |  |  | 137.7 (36%) | 132.1 (32.9%) |  |  | 137.7 (36.7%) | 130.3 (33.4%) |  |  |
| **Other** | 26.0 (6.6%) | 27.0 (6.9%) |  |  | 24.7 (6.5%) | 31.6 (7.9%) |  |  | 24.7 (6.6%) | 28.5 (7.3%) |  |  |
| **Admission period (year groups)** | | | | | | | | | | | | |
| **2016-2018** | 145.0 (37%) | 127.0 (32.3%) | 0.047 | 0.295 | 141.3 (37%) | 142.8 (35.6%) | 0.014 | 0.945 | 137.7 (36.6%) | 137.4 (35.2%) | 0.014 | 0.931 |
| **2019-2021** | 126.0 (32.1%) | 127.0 (32.3%) |  |  | 126.4 (33.1%) | 135.4 (33.7%) |  |  | 123.7 (32.9%) | 129.4 (33.2%) |  |  |
| **2022-2025** | 121.0 (30.9%) | 139.0 (35.4%) |  |  | 114.4 (29.9%) | 123.2 (30.7%) |  |  | 114.4 (30.4%) | 123.3 (31.6%) |  |  |
| **Laboratory parameters, median (IQR)** | | | | | | | | | | | | |
| **CRP, mg/L** | 5.00 (1.95, 5.00) | 5.00 (2.68, 5.81) | 0.334 | 0.003 | 5.00 (2.54, 5.12) | 5.00 (2.4, 5.00) | 0.056 | 0.729 | 5.00 (2.48, 5.00) | 5.00 (2.38, 5.00) | 0.073 | 0.874 |
| **WBC, 10^9^/L** | 7.40 (6.18, 8.80) | 6.70 (5.50, 8.10) | -0.170 | <0.001 | 7.40 (6.00, 8.90) | 7.00 (5.70, 9.00) | 0.209 | 0.440 | 7.40 (6.00, 8.80) | 7.00 (5.70, 8.70) | 0.105 | 0.206 |
| **RBC, 10^12^/L** | 4.66 (4.35, 5.00) | 4.59 (4.20, 4.95) | -0.131 | 0.063 | 4.59 (4.26, 4.96) | 4.63 (4.27, 4.99) | 0.024 | 0.705 | 4.60 (4.26, 4.96) | 4.63 (4.25, 4.98) | 0.018 | 0.783 |
| **PLT, 10^9^/L** | 218.00 (181.00, 256.50) | 194.00 (161.00, 233.00) | -0.231 | <0.001 | 208.00 (174.00, 246.00) | 206.00 (165.00, 248.00) | 0.061 | 0.647 | 208.00 (174.00, 247.00) | 204.00 (163.00, 246.00) | 0.001 | 0.302 |
| **Hb, g/L** | 143.00 (132.00, 154.00) | 139.00 (127.00, 151.00) | -0.210 | 0.002 | 141.00 (129.00, 152.00) | 141.00 (130.00, 153.00) | 0.024 | 0.996 | 141.00 (129.00, 152.00) | 141.00 (130.00, 152.00) | 0.011 | 0.896 |
| **HbA1c, %** | 5.90 (5.60, 6.30) | 6.20 (5.80, 7.70) | 0.639 | <0.001 | 6.00 (5.70, 6.70) | 6.00 (5.70, 6.60) | 0.066 | 0.990 | 6.00 (5.70, 6.70) | 6.00 (5.70, 6.60) | 0.105 | 0.698 |
| **TG, mmol/L** | 1.19 (0.87, 1.66) | 1.15 (0.84, 1.64) | -0.099 | 0.346 | 1.17 (0.82, 1.56) | 1.15 (0.86, 1.59) | -0.037 | 0.891 | 1.15 (0.82, 1.53) | 1.15 (0.86, 1.57) | -0.027 | 0.854 |
| **HDL-C, mmol/L** | 1.18 (0.98, 1.40) | 1.13 (0.94, 1.35) | -0.134 | 0.073 | 1.15 (0.95, 1.39) | 1.17 (0.99, 1.36) | 0.009 | 0.613 | 1.17 (0.96, 1.40) | 1.17 (0.99, 1.37) | -0.01 | 0.787 |
| **LDL-C, mmol/L** | 3.05 (2.46, 3.71) | 2.85 (2.27, 3.43) | -0.245 | <0.001 | 3.01 (2.40, 3.61) | 2.97 (2.41, 3.54) | -0.026 | 0.743 | 3.00 (2.39, 3.62) | 2.93 (2.41, 3.53) | -0.026 | 0.748 |
| **CRE, umol/L** | 76.00 (65.00, 89.00) | 80.00 (67.00, 102.00) | 0.194 | 0.002 | 78.00 (67.00, 93.00) | 79.00 (67.00, 99.00) | -0.005 | 0.598 | 78.00 (66.00, 92.00) | 79.00 (66.00, 98.00) | 0.008 | 0.498 |
| **ALB, g/L** | 42.85 (40.10, 45.42) | 41.60 (38.60, 44.10) | -0.168 | <0.001 | 42.10 (39.00, 44.80) | 42.10 (39.10, 44.50) | -0.037 | 0.958 | 42.10 (39.10, 44.80) | 42.00 (39.10, 44.50) | -0.046 | 0.723 |
| **Hcy, mmol/L** | 14.16 (10.54, 19.43) | 14.64 (11.14, 19.39) | -0.023 | 0.606 | 13.98 (10.59, 19.79) | 14.46 (11.22, 19.5) | -0.042 | 0.825 | 14.14 (10.66, 19.90) | 14.57 (11.36, 19.50) | -0.043 | 0.868 |

Note: After inverse probability weighting, the displayed counts represent weighted sample sizes and may therefore be non-integer values. Covariate balance was primarily assessed using standardized mean differences (SMDs). High and low GLR groups were defined according to the ROC-derived cutoff value of 4.137. Continuous variables are presented as median (interquartile range), and categorical variables as number (percentage). An SMD of less than 0.1 was considered indicative of adequate balance.

Abbreviations: GLR, glucose-to-lymphocyte ratio; IPW, inverse probability weighting; SMD, standardized mean difference.

**Supplementary Table 2. Logistic Regression Analysis of Quartiles of Glucose-to-Lymphocyte Ratio and Poor Functional Outcome.**

| **GLR quartiles** | **OR (95% CI)** | **P-value** |
| --- | --- | --- |
| Q1 | Reference | / |
| Q2 | 1.091(0.582~2.044) | 0.786 |
| Q3 | 1.858(1.001~3.451) | 0.050 |
| Q4 | 1.491(0.777~2.863) | 0.229 |
| P for trend | 1.058(0.959~1.166) | 0.262 |

Note: Poor functional outcome was defined as a modified Rankin Scale score of 3–6 at 90 days. GLR was categorized into quartiles, with the lowest quartile (Q1) as the reference group. P for trend was calculated by assigning the median GLR value of each quartile to all participants in that quartile and entering this variable as a continuous term in the adjusted logistic regression model. Odds ratios were estimated using logistic regression with the same covariate adjustment set as the main Model 2, including study period, sex, age, systolic blood pressure, diastolic blood pressure, onset-to-needle time, TOAST classification, previous stroke or transient ischemic attack, atrial fibrillation, diabetes mellitus, hypertension, coronary artery disease, smoking, baseline NIHSS score, HbA1c, CRP, WBC, RBC, Hb, PLT, CRE, Hcy, TG, HDL-C, LDL-C, and ALB.

**Supplementary Table 3. Sensitivity Analysis Using an Alternative Dichotomization of 90-Day Functional Outcome.**

| **GLR levels** | **OR (95% CI)** | **P-value** |
| --- | --- | --- |
| GLR | 1.014(0.954~1.078) | 0.655 |
| **GLR level, low vs high** | | |
| Low | Reference | / |
| High | 1.258(0.863~1.834) | 0.233 |

Note: In this sensitivity analysis, poor functional outcome was redefined as an mRS score of 2–6 at 90 days, and favorable outcome as an mRS score of 0–1. High and low GLR groups were defined according to the ROC-derived cutoff value of 4.137. Both analyses used the same covariate adjustment set as the main Model 2, including study period, sex, age, systolic blood pressure, diastolic blood pressure, onset-to-needle time, TOAST classification, previous stroke or transient ischemic attack, atrial fibrillation, diabetes mellitus, hypertension, coronary artery disease, smoking, baseline NIHSS score, HbA1c, CRP, WBC, RBC, Hb, PLT, CRE, Hcy, TG, HDL-C, LDL-C, and ALB.
